# Implementation and performance of SIBYLS: a dual endstation small-angle X-ray scattering and macromolecular crystallography beamline at the Advanced Light Source

**DOI:** 10.1107/S0021889812048698

**Published:** 2013-01-17

**Authors:** Scott Classen, Greg L. Hura, James M. Holton, Robert P. Rambo, Ivan Rodic, Patrick J. McGuire, Kevin Dyer, Michal Hammel, George Meigs, Kenneth A. Frankel, John A. Tainer

**Affiliations:** a Physical Bioscience Division, Lawrence Berkeley National Laboratory, Berkeley, CA 94720, USA; b Life Sciences Division, Lawrence Berkeley National Laboratory, Berkeley, CA 94720, USA; c Department of Biochemistry and Biophysics, University of California, San Francisco, CA 94158-2330, USA; d Engineering Division, Lawrence Berkeley National Laboratory, Berkeley, CA 94720, USA

**Keywords:** small-angle X-ray scattering (SAXS), macromolecular crystallography (MX), synchrotron beamlines, SIBYLS

## Abstract

The SIBYLS beamline (12.3.1) of the Advanced Light Source at Lawrence Berkeley National Laboratory, supported by the US Department of Energy and the National Institutes of Health, is optimized for both small-angle X-ray scattering (SAXS) and macromolecular crystallography (MX), making it unique among the world’s mostly SAXS or MX dedicated beamlines. Since SIBYLS was commissioned, assessments of the limitations and advantages of a combined SAXS and MX beamline have suggested new strategies for integration and optimal data collection methods and have led to additional hardware and software enhancements. Features described include a dual mode monochromator [containing both Si(111) crystals and Mo/B_4_C multilayer elements], rapid beamline optics conversion between SAXS and MX modes, active beam stabilization, sample-loading robotics, and mail-in and remote data collection. These features allow users to gain valuable insights from both dynamic solution scattering and high-resolution atomic diffraction experiments performed at a single synchrotron beamline. Key practical issues considered for data collection and analysis include radiation damage, structural ensembles, alternative conformers and flexibility. SIBYLS develops and applies efficient combined MX and SAXS methods that deliver high-impact results by providing robust cost-effective routes to connect structures to biology and by performing experiments that aid beamline designs for next generation light sources.

## Abstract 2

The SIBYLS beamline of the Advanced Light Source at Lawrence Berkeley National Laboratory is a dual endstation small-angle X-ray scattering and macromolecular crystallography beamline. Key features and capabilities are described along with implementation and performance.

## 1. Introduction

The SIBYLS beamline (structurally integrated biology for life sciences) produced its first structure in 2004 (Barondeau *et al.*, 2004 ▶) and became available to general users in 2005 (Trame *et al.*, 2004 ▶). From the outset, it was envisaged as a highly configurable structural biology beamline: a tool for tackling difficult problems in biology exemplified by large, dynamic and not-easily crystallized macromolecules. By offering both solution scattering and macromolecular crystallographic capabilities, SIBYLS was designed to leverage the individual strengths of each technique and combine them to reveal new insights into the structure, and ultimately the function and mechanism, of challenging biological systems. This vision has been borne out in the subsequent years with many high-impact papers published that incorporate both small-angle X-ray scattering (SAXS) and macromolecular crystallography (MX) results (Hammel *et al.*, 2011 ▶; Williams *et al.*, 2011 ▶; Nishimura *et al.*, 2009 ▶; Williams *et al.*, 2009 ▶). The trend in macromolecular crystallography, and more generally in bioscience research, is towards automated and high-throughput methods. There have been numerous efforts from both SAXS (Round *et al.*, 2008 ▶; David & Perez, 2009 ▶; Blanchet *et al.*, 2012 ▶) and MX (Karain *et al.*, 2002 ▶; Snell *et al.*, 2004 ▶; Cipriani *et al.*, 2006 ▶; Cork *et al.*, 2006 ▶; Soltis *et al.*, 2008 ▶; Jacquamet *et al.*, 2009 ▶; Smith *et al.*, 2010 ▶; Murakami *et al.*, 2012 ▶) beamlines to implement high-throughput and automated data collection methods. The SIBYLS beamline is heavily oversubscribed because of the rising use of biological SAXS. We have, therefore, made many improvements to the SIBYLS beamline in the past several years to increase the efficiency and throughput of this valuable resource while at the same time maintaining and improving the accuracy and precision of the beamline hardware and software (Hura *et al.*, 2009 ▶; Classen *et al.*, 2010 ▶). This manuscript will describe the major features of the SIBYLS beamline, some of the more recent advancements in hardware and software, and scientific highlights.

## 2. Optics

**Figure 1:**
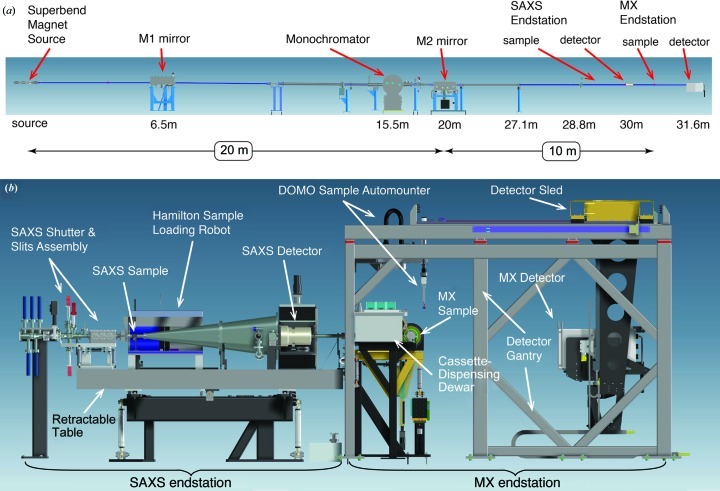
SIBYLS optics and equipment overview. (*a*) Schematic diagram of the SIBYLS beamline showing all optical elements from the 5 T superbend magnet to the MX detector. (*b*) Side view of the dual SAXS and MX endstations with major components labeled.

The X-ray source for SIBYLS is a 5.0 T superconducting bending magnet with a critical energy of 12 keV – one of three superconducting 5.0 T superbend magnets that replaced the preexisting normal conducting 1.3 T bending magnets (Robin *et al.*, 2002 ▶). The upgrade was implemented during two six-week shutdowns in 2001 and has subsequently allowed the development of techniques at the Advanced Light Source (ALS) that are dependent on hard X-rays, such as tomography, powder diffraction, macromolecular crystallography and small-angle X-ray scattering. The superbend magnets are located at sectors 4, 8 and 12. A suite of three mini-hutch MX beamlines was developed at sector 8 (MacDowell *et al.*, 2004 ▶), which laid the groundwork for the development of the SIBYLS beamline at sector 12. Whereas the sector 8 MX beamlines use mini-hutches, SIBYLS was designed to accommodate interchangeable SAXS and MX endstations and it was decided to enclose the entire experimental station within a large walk-in hutch to provide flexibility in experimental design beyond the capabilities of the mini-hutches (Fig. 1 ▶).

The basic beamline optics design (Table 1 ▶) is similar to the other MX superbend beamlines at the ALS (MacDowell *et al.*, 2004 ▶; Trame *et al.*, 2004 ▶). Directly after the 5.0 T superbend is the M1 mirror, a vertically deflecting plane paraboloid collimating mirror (grazing angle = 4.5 mrad, acceptance = 1.5 × 0.5 mrad) that provides parallel radiation for the monochromator (§2.1), followed by a toroidal mirror (M2) that focuses the light into the hutch on either the SAXS endstation (where it is focused at the beamstop) or the MX endstation (where it is typically focused at the sample position). The SAXS endstation is upstream and in series with the MX endstation. The SAXS endstation is on a translatable Newport optical table and can be inserted or retracted to allow for operation of the SAXS or MX endstation, respectively (§5). The change in focus position is achieved by adjusting the bending radius of the M2 mirror along with its tilt angle. The M1 mirror is a flat internally water cooled electroless nickel-plated Invar mirror. It is held in a mechanical bender fixed at the required parabolic shape. The M2 mirror is an uncooled silicon cylindrical mirror bent into a toroid. Both mirrors are coated with 8 nm of rhodium over 25 nm of platinum.

### 2.1. Monochromator

**Figure 2:**
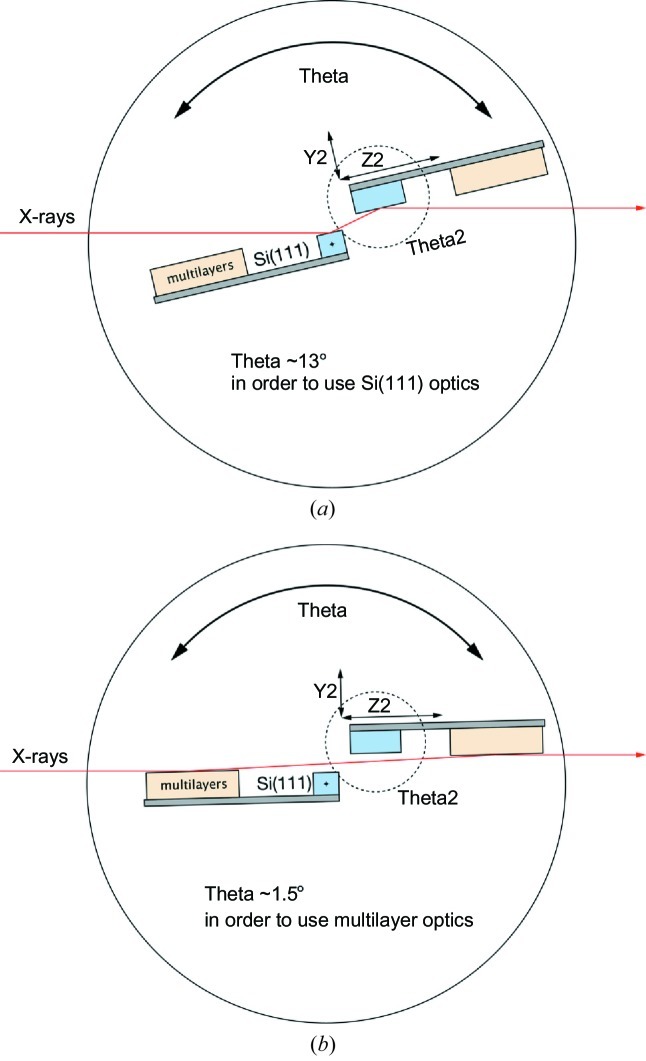
Schematic diagram illustrating the custom Si(111)/multilayer monochromator. (*a*) Rotation of the main θ stage to ∼13° allows use of Si(111) crystals. (*b*) Rotation of the θ stage to ∼1.5° allows use of the multilayer optical elements.

The monochromator is a customized Kohzu APM-type monochromator offering both Si(111) crystals and multilayer optics (Fig. 2 ▶). The Si(111) setup is identical to that described by MacDowell *et al.* (2004 ▶). Both multilayer elements are flat unbent elements with 150 layer pairs of Mo/B_4_C with *d* spacing = 2.4 nm (Osmic Inc., Troy, MI, USA). The first multilayer is side cooled by water. The second Si crystal and multilayer are uncooled elements. The monochromator rotates all internal optics about the central θ axis, which is coincident with the reflecting surface of the first Si crystal. The second Si crystal is mounted on a stack of four motorized stages, which in turn is supported from the main θ rotation platform. The second crystal is adjusted to maintain a constant beam height exiting the monochromator. The first multilayer is fixed to the main θ support structure and mounted upstream of the first Si crystal. The second multilayer is mounted on the same stage as the second Si(111) crystal. Because the surface of the first multilayer element is not positioned on the main θ axis the θ angle must be moved to very low angle so that it will intercept the incoming beam. The beam is then directed to the second multilayer (Fig. 2 ▶). The shallow θ angle required for the multilayer elements results in the X-ray beam drifting off the surface as the energy is changed, but by a suitable choice of mirror length (176 mm) and *d* spacing (2.4 nm) it is possible to achieve a useful energy range of ∼7–13 keV. The benefit of the multilayer optics is to increase the flux (∼40-fold) at the expense of higher band pass [*E*/Δ*E* is 7000 for Si(111) and 110 for the multilayers].

### 2.2. Beam conditioning slits

Between the monochromator and the hutch are two sets of slits. The first set, directly before the M2 mirror tank, defines the horizontal and vertical convergence angles of the beam. The convergence of the full beam after the M2 mirror is 3 mrad (horizontal, H) × 0.3 mrad (vertical, V). The X-ray beam diverges in the horizontal plane until the M2 mirror, but the M1 mirror collimates the beam in the vertical plane. This collimation is necessary because a significant spread in vertical incidence angles would degrade monochromator performance, but the effect of a horizontal spread is negligible. Because of Bragg’s law, a range of vertical incidence angles results in a range of wavelengths to all be emitted in the same direction by the first crystal and then in different directions by the second crystal. The result is similar to that of a thermal bump or other distortion on the crystal quality: increased bandwidth and reduced flux onto the sample. The performance of the monochromator was tested by evaluating the width of the extremely narrow white line in the XANES spectrum from Krypton dissolved at high pressure in Paratone-N oil. It was found to be 2 eV wide at 14 keV, or exactly the 7000:1 ratio expected for Si(111).

The second set of slits are used only for SAXS experiments and provide the first (of three) guards against parasitic scattering. These slits are moved out of the beam when in MX mode. In order to achieve the cleanest possible beam for SAXS experiments with no parasitic scattering, two additional sets of slits are located just before the SAXS sample position. After switching to SAXS mode these are manually adjusted to reduce parasitic scattering.

## 3. SAXS endstation

The SAXS station shares all X-ray optics with the MX endstation. When the SAXS endstation is inserted, X-rays pass through the final SAXS slits and the helium-containing shutter box to reach the SAXS sample cell and detector (Fig. 1 ▶). Most hardware that requires movement has been motorized and is controlled *via* the dedicated *Blu-Ice* and *DCS* system originally developed at the Stanford Synchrotron Radiation Lightsource (SSRL; McPhillips *et al.*, 2002 ▶) and modified specifically for SAXS data collection at the SIBYLS beamline (Classen *et al.*, 2010 ▶). Available equipment includes a fast experimental shutter (capable of 50 ms exposures), an active video feedback system for beam stabilization, a sample-visualization system, an automated liquid-sample-loading system based on an ML4000 liquid-handling robot (Hamilton Company, Reno, NV, USA), and a highly configurable sample cell. To achieve sufficient flux for rapid data collection and informative scattering from samples with low concentrations and small volumes, we primarily use the multilayer optics of the monochromator. The tunable-wavelength X-rays enable rapid adjustment of the scattering vector magnitude (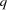) appropriate for the experiment without changing the sample-to-detector configuration [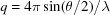, where θ is the scattering angle and λ is the wavelength]. Scattering is measured on a mar165 area detector (Rayonix, Evanston, IL, USA), coaxial with the incident beam and positioned 1.5 m from the sample, allowing measurement of 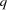 ranges from a minimum of 0.007 Å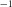 to a maximum of 4.2 Å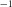.

### 3.1. SAXS sample cell

**Figure 3:**
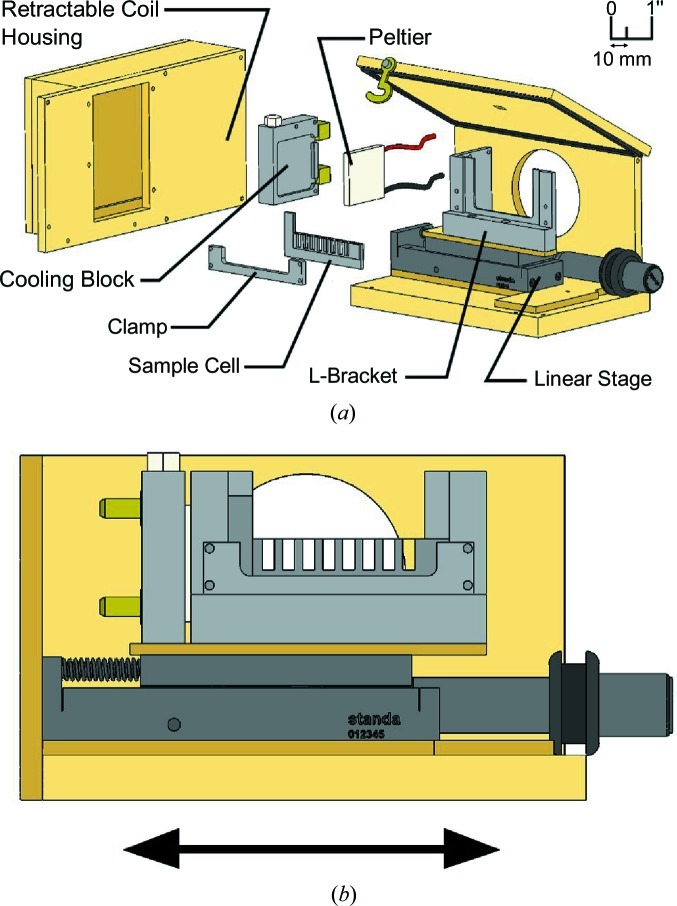
SAXS sample holder. (*a*) Exploded diagram of the SAXS sample cell showing the major components and (*b*) a front view showing the direction of motion of the multi-well sample stage. The front and right sides have been omitted for clarity.

Unlike MX where samples are maintained at cryogenic temperatures (93 K), SAXS experiments are performed on liquid samples. To address this issue we have designed a static multiple-well sample holder that accommodates a range of X-ray energies, minimizes background scatter, regulates both sample temperature and atmospheric conditions (aerobic *versus* anaerobic), and facilitates automation. The sample holder was modified from the design of Hiro Tsuruta (Tsuruta & Johnson, 2001 ▶). An exploded view is shown in Fig. 3 ▶. The L-bracket, cooling block, clamp and sample cell are aluminium, which is easily machinable and has excellent thermal conductivity. The cooling block contains internal channels connected to a circulating chilled water supply. A thermoelectric Peltier unit able to maintain sample temperatures from 277 to 363 K sits between the cooling block and the sample L-bracket. The sample cell temperature is monitored with a thermocouple directly attached to the sample cell. The multi-well sample cell contains eight wells that are 3.2 mm wide, permitting X-rays to pass through a large cross section of the sample before coming to a focal point 1.5 m downstream at the beamstop. The windows for the sample cell are fabricated from 25 µm-thick potassium aluminosilicate (muscovite mica) sheets (Goodfellow, USA). Mica was chosen because of its negligible contribution to background scattering. A single front and back window are cut and affixed to the aluminium sample cell using ÅngströmBond EPO-TEK 302-3M epoxy (Fiber Optic Center, New Bedford, MA, USA). The L-bracket is bolted to the linear stage (Standa Ltd), and is separated with a thin insulating piece of acrylic. The entire sample holder is enclosed in a sealed helium-purged acrylic box. In addition to permitting anaerobic experiments, the dry helium environment decreases background scatter relative to the atmosphere and prevents the accumulation of condensation, resulting in better quality data. The top of the box has hinges for quick access. A single 1 × 4 mm slot on the top of the lid, centered directly above the sample cell, allows the Hamilton robot (§3.2) to access the sample cell. This small opening is the only one in the sample cell holder and by maintaining a slight positive pressure of helium an oxygen-free atmosphere is maintained (see supplementary information^1^ for details of the oxygen-free verification experiment).

### 3.2. SAXS sample automation

**Figure 4:**
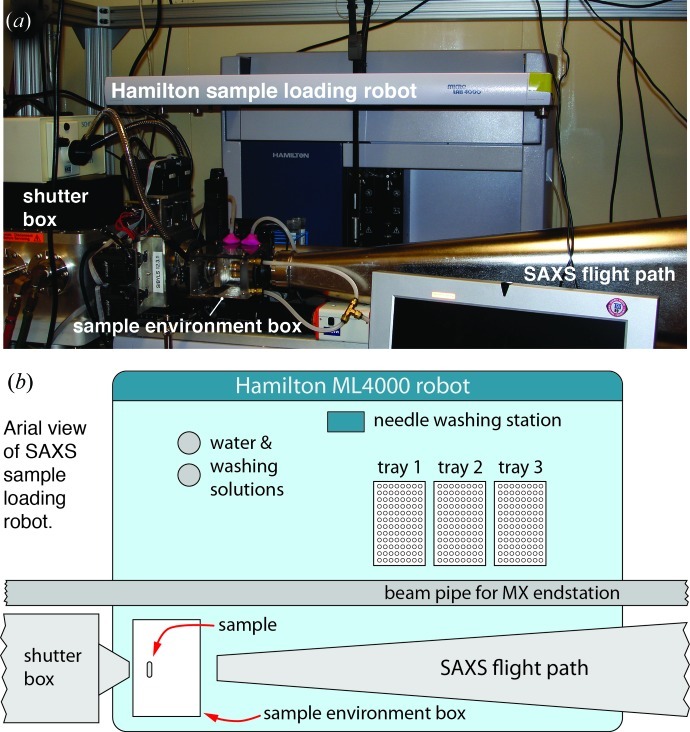
Hamilton ML4000 liquid-handing robot. (*a*) SAXS endstation showing the sample cell, Hamilton sample-loading robot and part of the evacuated flight tube leading to the detector. (*b*) Top schematic view of the Hamilton robot showing the relative positions of the 96-well sample plates, the sample cell and the other endstation components.

A Hamilton ML4000 liquid-handing robot (Fig. 4 ▶) loads and unloads samples without users needing to enter the hutch, significantly reducing data collection times and decreasing the number of user errors. After samples are prepared in 96-well plates, the entire SAXS experiment can be conducted remotely from the computer work station in a manual mode or by using the fully automatic hands-off screening functionality of *Blu-Ice*. Pulldown menus allow users to select the desired sample volume and aspiration rate (*e.g.* slower for more viscous samples). After entering the sequence of wells containing samples and buffers, up to five exposure times can be set, as well as five preprogrammed Hamilton robot commands (*e.g.* load sample, wash with water, empty to garbage, empty to well, remove bubble *etc.*). The robot loads sample solutions from the 96-well plate directly to the sample cell for exposure, returns the solutions back to the 96-well plate, and then rinses and washes the sample cell. These tasks are carried out with a speed impossible to achieve manually because radiation safety interlocks would need to be repeatedly set and unset for each activity. The numbers of buffers and concentrations collected per sample are fully configurable. For example, the collection of 48 samples and 48 alternating buffers, a full 96-well plate, can be completed in about 4 h.

## 4. MX endstation

The MX endstation shares all X-ray optics with the SAXS endstation. When the SAXS station is retracted a straight section of vacuum pipe sealed at each end with 50 µm beryllium windows is inserted to allow the X-rays to reach the MX station (see §5). All hardware that requires movement has been motorized and is controlled *via* the *Blu-Ice*/*DCS* system. Equipment available at the MX station includes a fast experimental shutter (capable of 50 ms exposures), an active video feedback system for beam stabilization, an on-axis sample-visualization system, a Huber XYZ sample stage (Huber 5102.05 XY-Stage, Huber 5104.B10 Z-Stage) and an air bearing (Fox Instrument and Air Bearing, Livermore, CA, USA) for rapid ϕ-axis rotation. The air bearing enables ‘round robin’ MAD data collections where the crystal is flipped 180° after every image and the photon energy is changed every other image to measure both anomalous and dispersive differences as close in time as possible. Additional equipment includes a retractable Evex silicon drift diode fluorescence detector (Evex, Princeton, NJ, USA) with 128 eV energy resolution connected to a DSA-1000 multi-channel analyzer (Canberra Industries Inc., Meriden, CT, USA), user adjustable scatterless slits to define beam size on the sample, a cryogenic cold stream system, an automated sample-mounting system adapted from the SSRL-style SAM automounter, and an ADSC Quantum 315r CCD detector (mounted on a robust gantry system capable of 2θ offsets from −5° up to +45° and distances from 120 to 1600 mm). Details of some of these key features are described below.

### 4.1. Fast experimental shutter

The shutter is a model LS055 from NM Laser (San Jose, CA, USA). The same model is used for both the SAXS and the MX endstations. We have measured the jitter of these shutters, and it is ∼0.6 ms r.m.s. and dominated by the electronics, not the mechanics. The shutter and spindle are synchronized by a PMAC (Delta Tau Inc., Chatsworth, CA, USA) motion controller running a control program every 2 ms. This program actuates the shutter when the spindle encoder has passed the desired opening or closing positions, generating a sawtooth-shaped distribution of opening-time errors. The r.m.s. variation of a sawtooth with 2 ms period is 0.577 ms, and the average error of two shutter events is therefore expected to be 0.41 ms. This is remarkably consistent with the 0.47 ms r.m.s. timing error inferred from the variation in the refined miss-setting angle about the spindle axis observed in MX data taken with 0.1 s exposure times. In the absence of sample drift, the miss-setting angle in this direction divided by the rotation speed is the average error of two shutter events, indicating that the 2 ms execution time of the programmable logic controller is the dominant source of error in shutter timing. For this reason, users are advised to keep their exposures above 50 ms so that the shutter does not introduce more than 1% error into partially recorded reflections.

### 4.2. Beam positioning

The focused uncollimated beam size at the sample is 165 × 130 µm FWHM (H × V). The user-adjustable slits (§4.4) enable the horizontal and vertical dimensions to be independently adjusted from fully open to fully closed. During an experiment the beam must remain fixed on the sample for long periods of time, over which we observe slow thermal variations of optical supports and other environmental changes that cause fluctuations in beam position. When the user defines a small beam with the adjustable slits the problem of drift is less severe, because the beam overfills the limiting aperture directly before the sample, but it is still important to maintain a consistent beam position. Similar to other MX beamlines at the ALS, we have implemented a video feedback system for maintaining a stable beam position (MacDowell *et al.*, 2004 ▶). A 50 µm-thick cerium-doped yttrium aluminium garnet (YAG; Startech Instruments, New Fairfield, CT, USA) is glued to the upstream side of the shutter blade located 170 mm before the sample in a helium-filled aluminium box that contains the experimental shutter and an ion chamber to measure beam flux. The shutter blade is inclined by 7° relative to the X-ray beam, and operates by flipping the blade in the vertical: like a diving board. X-rays cause the YAG to luminesce and the image is monitored by a CCD camera positioned on top of the helium box looking down through a viewport at the shutter. After a full tune-up of the beamline optics the centroid of the luminescing YAG image is recorded and the video feedback system makes small adjustments to the pitch of the second mirror (M2 tilt) and the roll of the second monochromator crystal (Chi2) to move the beam in the vertical and horizontal directions, respectively. The feedback system maintains a stable beam for hours, even with frequent monochromator energy changes. An identical feedback system also improves the beam quality for the SAXS experiments by maintaining a steady beam position and preventing the beam from drifting into the guard slits, which would generate undesirable scatter.

### 4.3. DOMO (dynamic offsite MX operator)

**Figure 5:**
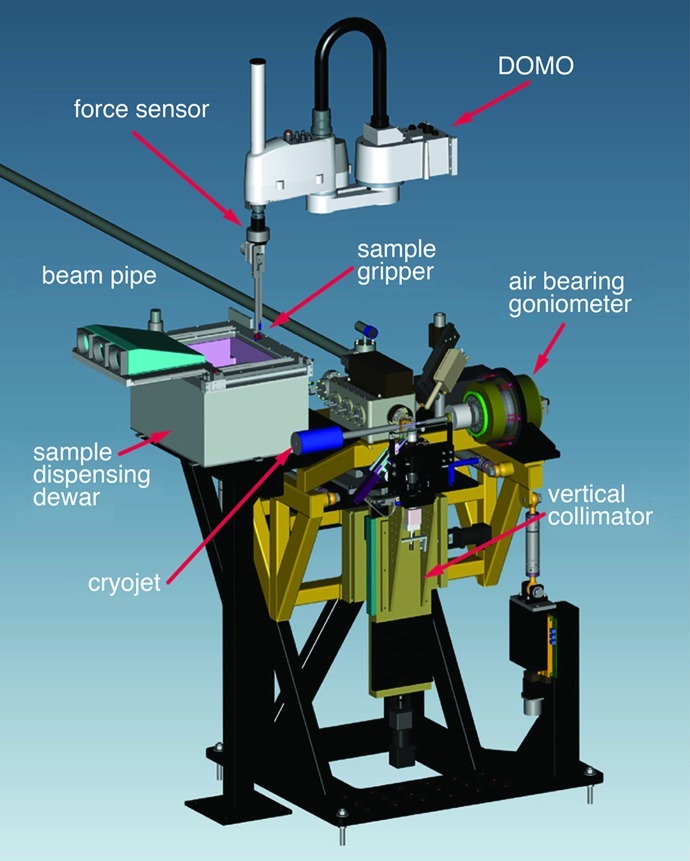
MX endstation showing key features of the sample-positioning system. The DOMO automated MX sample-loading robot is colored white. The supporting gantry has been omitted for clarity. The lid of the sample-dispensing dewar is shown in the open position.

Our automated sample-mounting system, DOMO, was adapted from the SSRL SAM design with modifications to fit within the constraints of our MX station. The basic SAM system (Cohen *et al.*, 2002 ▶) is based on a commercial Epson SCARA robot arm (model E2S453SM, EPSON Robots, Carson, CA, USA). A further modification to the SAM design was the addition of a sliding-lid sample-dispensing dewar (Fig. 5 ▶). The sliding lid minimizes vibration associated with a hinged clam-like lid as well as the production and precipitation into the dewar of the ice that forms when warm humid air mixes with cold nitrogen gas. The sample dewar holds two 96-port SSRL-style sample cassettes (Crystal Positioning Systems, Jamestown, NY, USA) for a total capacity of 192. DOMO is fully compatible with SSRL cassettes already in circulation. The sample-dispensing dewar can also hold two uni-puck adapters which accommodate eight uni-pucks, for a total capacity of 128 samples. In addition to mounting and dismounting crystals, users can command DOMO to wash adherent ice off of their samples from the *Blu-Ice* interface. DOMO is equipped with a multi-axis force sensor to facilitate robot alignment tasks. At the beginning of each MX session automated procedures calibrate the magnetic picker/placer tool, the sample cassettes and the goniometer. The force sensor is also used to probe individual samples in the cassette to determine if they are loaded improperly or if they have ice on the bottom that will interfere with proper robot operation. Implementation of DOMO at the SIBYLS MX station has enabled higher throughput and greater user accessibility and is a key feature of our remote MX data collection program (§7.2).

### 4.4. Scatterless slits

A recent upgrade to the MX endstation involved replacement of the fixed-diameter tantalum pinhole system (consisting of interchangeable 30, 50 and 100 µm pinholes) with a user-adjustable piezo-actuated hybrid tantalum metal/single-crystal slit system. Our design was inspired by similar scatterless slits developed for SAXS experiments that use silicon or germanium crystals for the slit edges (Li *et al.*, 2008 ▶). One significant difference is our choice of single tantalum crystals for the slit edges over silicon or germanium, as the former provide superior attenuation of X-rays in the energy range used for MX. The slits allow the user to independently adjust the size of the X-ray beam on the sample from 10 to 130 µm in both the horizontal and vertical directions. This is useful because matching the beam size to the crystal size results in an improved diffraction limit, lower mosaicity, a lower *R*_merge_ and a better signal-to-noise ratio for the data (Sanishvili *et al.*, 2008 ▶). Sanishvili and co-workers also showed that a small beam allows users to selectively irradiate smaller better-diffracting regions of a larger imperfectly diffracting crystal. Note that this system provides a ‘mini beam’ where small beam sizes are achieved not by focusing but rather by using slits to reduce the focused beam profile down to the desired size. For beam sizes down to 10 µm the slits may change the flux (photons per second) but do not change the flux density (photons per area per second) and, therefore, also do not change the crystals’ useful lifetime in the beam. They will still reach the Owen *et al.* (2006 ▶) damage limit (30 MGy) in about 30 min at the SIBYLS beamline (Holton, 2009 ▶; Holton & Frankel, 2010 ▶).

### 4.5. Sample visualization

The MX endstation is equipped with both low- and high-magnification on-axis sample-viewing systems. Initial sample alignment is done at low magnification. This has the advantage of making high-magnification alignment fairly easy, and the sample can be quickly positioned in the cryostream. The on-axis sample viewing is particularly critical for alignment of very small crystals. A 5 × 5 mm 45° mirror, with a 0.8 mm-diameter hole drilled through to allow X-rays to pass, is positioned 8 mm before the sample and reflects visible light from the sample to a 10× long-working-distance microscope objective (Mitutoyo M Plan Apo 378-803-3), with a field of view on the CCD camera of 580 × 460 µm. Diffuse low-coherence back illumination is provided by focusing a fiber-optic illuminator at the strip of white polyethylene foam that supports the back stop. The high-magnification microscope, the back stop and the adjustable slits are mounted on a large XY stage (see ‘vertical collimator’ in Fig. 5 ▶) that lowers them ∼180 mm out of the way prior to sample mounting. This feature allows the goniometer spindle to be completely accessible to the DOMO sample-mounting robot without disturbing the delicate components of the optical system, the back stop or the adjustable slits. With the stage lowered, a large 45° mirror, located on the front of the helium-filled shutter box, captures the sample image on-axis and reflects it to the low-magnification microscope, an Infinity Optics K2/SC long-working-distance microscope (Infinity Photo-Optical, Boulder, CO, USA) with a field of view on the 1/2 inch CCD camera of 4.36 × 3.28 mm. This view allows for coarse alignment of the sample after initial mounting. When the vertical collimator stage is raised, the sample is viewed with the higher-magnification system for more precise final alignment.

### 4.6. Control system

The MX endstation uses the *Blu-Ice*/*DCS* control system originally developed at SSRL (McPhillips *et al.*, 2002 ▶; Soltis *et al.*, 2008 ▶) because of the modular design of *DCS* (distributed control system), the ease of customizing the *Tcl*/*Tk*-based *Blu-Ice* graphical user interface (GUI), and the ability to quickly and easily write new DHS (distributed hardware server) modules for new hardware. Briefly, *DCS* is the central hub through which all commands are sent and complex operations are coordinated, DHSs allow specific pieces of hardware to communicate with *DCS*, and *Blu-Ice* is the GUI for users and beamline scientists to control the entire beamline. The SAXS endstation also uses *Blu-Ice*/*DCS*, although it has been heavily modified for the specific requirements of collecting SAXS data (Classen *et al.*, 2010 ▶). It is a testament to the flexibility and robustness of the SSRL *Blu-Ice*/*DCS* system that it has been so easily integrated into an independent beamline design. The details of the *Blu-Ice* GUI have been thoroughly described elsewhere (McPhillips *et al.*, 2002 ▶; Soltis *et al.*, 2008 ▶). For the MX endstation the most significant changes to the *Blu-Ice*/*DCS* code have been to modify features unique to SIBYLS (*e.g.* our 2θ offset *versus* a simple detector translation at SSRL and our two-cassette sample-dispensing dewar *versus* the SSRL three-cassette dewar). Additionally, many motors are controlled by the underlying *LabVIEW* system, which required the development of *LabVIEW*-specific ‘thin’ DHSs to translate commands back and forth between *DCS* and *LabVIEW*.

## 5. Switching between endstations

**Figure 6:**
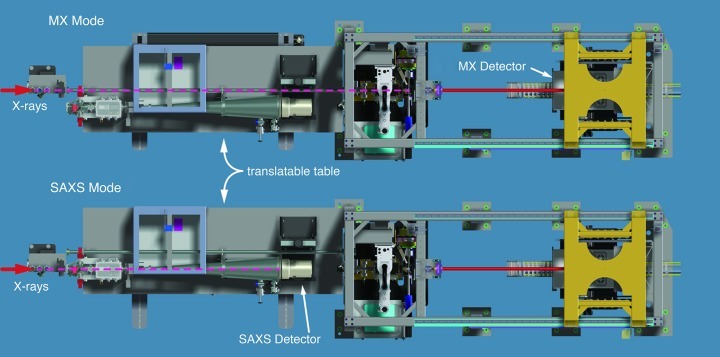
Schematic of the SIBYLS beamline. Top views showing the beamline in MX mode and SAXS mode. Conversion is accomplished by translated the SAXS support table by ∼20 cm. The path of the X-rays is shown by a dashed line.

The SIBYLS beamline was designed for quick and easy conversion between SAXS and MX modes. Briefly, the steps involved to switch from MX to SAXS or *vice versa* are as follows: (1) Insert or retract the SAXS station by translating the Newport support table (Fig. 6 ▶) by ∼20 cm. (2) Execute a changeover script which moves the shared beamline optical elements to values optimized for SAXS or MX. (3) Confirm the focus of the beam and perform a final automated tune-up. Switching from SAXS to MX takes about 10–20 min and going from MX to SAXS about 30 min. The MX to SAXS changeover is a little slower because the guard slits need to be manually positioned. Typically we alternate a week of SAXS and a week of MX. There are occasions when a single user needs to collect both SAXS and MX data during the same shift on the same sample, and we will switch the beamline from SAXS to MX during their shift, but this is the exception rather than the rule. Crystals can be flash-cooled and data collection scheduled to make it feasible for experimenters to obtain SAXS data from comparable solution samples. This strategy further facilitates combining solution conformation and assembly information from SAXS with precision information from MX.

## 6. Wet lab

In addition to the sample preparation bench at the beamline, users have access to a fully equipped biochemistry laboratory located within minutes of the beamline. The laboratory contains chromatographic purification and analysis equipment including ÄKTA Explorer and Ettan FPLC systems (GE Healthcare Biosciences, Pittsburgh, PA, USA) and a Bio-Rad chromatography system (Bio-Rad, Hercules, CA, USA). These systems can be coupled to an 18-angle multi-angle light-scattering detector integrated with a quasi-elastic light-scattering system for accurate mass and polydispersity measurements (Wyatt Technology Corporation, Santa Barbara, CA, USA). The laboratory also has a crystallization incubator and visualization system, various centrifuges, a NanoDrop UV/VIS spectrophotometer, and gel boxes for protein and nucleic acid separation. There is access to an autoclave and a walk-in 277 K cold laboratory in the building. The proximity of this equipment to the beamline is ideal for obtaining high-quality monodisperse SAXS data and for exploring various buffer conditions and ligand binding.

## 7. Remote and mail-in data collection

In the constant pursuit of a better, easier to use beamline and because of the ever increasing demand for both MX and SAXS beamtime, we have implemented high-throughput, remote and mail-in data collection modes.

### 7.1. Mail-in SAXS data collection

Automated liquid-handling systems have been installed at several synchrotron SAXS beamlines, including X33 of EMBL (Round *et al.*, 2008 ▶; Blanchet *et al.*, 2012 ▶), 4–2 at the SSRL, SWING at SOLEIL (David & Perez, 2009 ▶) and the SIBYLS beamline at the ALS (Hura *et al.*, 2009 ▶; Classen *et al.*, 2010 ▶). In all cases, the automation of sample delivery has vastly improved efficiency. Installation of the Hamilton automatic sample-loading system at the SIBYLS beamline (see §3.2) has reduced data collection time from ∼75 min per sample (two buffer blanks and three concentrations) to 7 min. This has enabled much higher data collection rates, resulting in the ability to accommodate more users within the standard 8 h shift. Most investigators bring 96-well trays to SIBYLS and exclusively use the liquid-handling system. This has had the unintended effect that investigators do not use their entire 8 h shift. Additionally, the beamline interface has grown necessarily complex and training new users requires significant time. To further increase efficiency we have implemented a mail-in/hand-in SAXS data collection program.

The principal objective for the mail-in/hand-in program is to provide high-quality SAXS data that are comparable to or better than data collected by users visiting the beamline in person. The mail-in/hand-in program has been operational since November 2010. Samples are typically sent preloaded in 96-well plates packed on wet ice. We have found that freezing-induced aggregation is highly sample dependent. This is easily tested before sending samples; many samples show no aggregation after freezing. If there is a minor level of aggregation, for example, stoichiometry and relative orientation of domains can still be determined. International shipping generally takes two–three days, which makes it feasible to send samples packed on wet ice to prevent freezing but still maintain cool temperatures. In rare cases of aggregation-prone samples, users are offered the option of sending concentrated frozen samples which are then thawed and serially diluted at the beamline prior to data collection.

**Figure 7:**
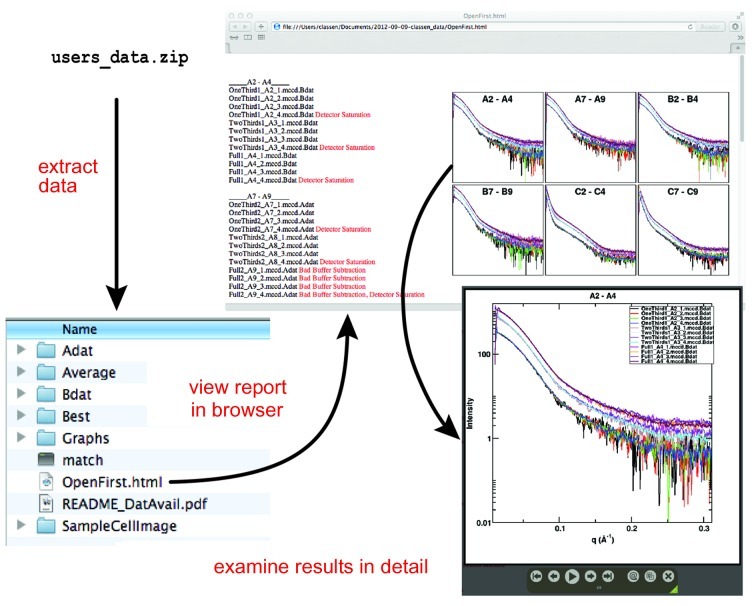
Example of the SAXS data package sent to mail-in/hand-in users. After extracting the data package, users can browse their results in the electronic HTML report. Investigators can click on each graph for an enhanced view of the scattering profile. Potentially problematic data sets are tagged by beamline staff and explanatory notes added.

Coordinating the arrival of samples with available beamtime is a challenge. Our solution has been to give users a window of time in which their samples should arrive. We then block out specific SAXS shifts dedicated to mail-in/hand-in data collection, and beamline staff collect data as soon as possible after the samples arrive. By doing so we provide flexibility to users who are preparing and shipping fresh samples while also making maximal use of beamtime. The Hamilton liquid-handling robot in its current configuration accommodates three 96-well plates, which take ∼12–13 h to collect. This allows beamline staff to queue enough samples to use the entire night shift. Users are then provided with raw data, processed data and a detailed report in PDF format (Fig. 7 ▶).

### 7.2. Remote MX data collection

MX users come from a wide geographical area, and travel is time consuming, inconvenient and expensive. Remote data collection offers convenience, efficient use of beamtime, flexibility in scheduling and greater access for a broader set of researchers, including students and postdocs (Smith *et al.*, 2010 ▶; Soltis *et al.*, 2008 ▶). Our solution has been to couple robotic mounting hardware (DOMO, §4.3) with remotely accessible software control of the beamline (*Blu-Ice* GUI connected *via* an NX Client). Since installation in 2008, DOMO has gained an extensive user base. Approximately 80% of MX shifts make use of DOMO, which has also proven its utility even when used by local users because it decreases sample-mounting/unmounting times, eliminates accidental damage to endstation components by users while they are in the hutch, and allows users to focus efforts on collecting and processing their data.

## 8. Exemplary scientific highlights and discussion

The two overall goals for the SIBYLS beamline are to solve structures informing cell biology and to aid integration of X-ray scattering and X-ray diffraction technologies to provide accurate conformations, structures and assemblies in solution under near physiological conditions. The beamline was also designed to be flexible and efficient to make maximal use of precious biological samples, which requires expert staff with a sustained and enthusiastic dedication to quality and ongoing improvements. Our experience indicates that a dual endstation beamline provides a more effective and productive work environment by providing sufficient time for thoughtful analysis and improvements by SAXS and MX scientists when the beamline switches to the alternative technique. The exemplary results noted here highlight the productivity of this combination: how seamlessly the MX and SAXS can each function productively on a multipurpose beamline, the added value from combined experiments, and the likely impact of beamlines such as SIBYLS relative to the investment required for optimal dedicated SAXS facilities.

### 8.1. SAXS results

Structural investigations, especially of macromolecules from higher eukaryotes, often result in partial structural information owing to the presence of intrinsically unfolded domains that are recalcitrant to traditional X-ray crystallography. At SIBYLS we have found that SAXS can provide structural information of the complete protein or complex, thus complementing partial high-resolution structural information from NMR or MX (Putnam *et al.*, 2007 ▶).

At the SIBYLS beamline SAXS investigations have shed light on samples ranging in size from water (Clark *et al.*, 2010 ▶) up to large protein–nucleic acid complexes such as DNA–PK/Ku/DNA (Hammel, Yu, Mahaney *et al.*, 2010 ▶), the RNA chaperone FinO with RNA (Arthur *et al.*, 2011 ▶) and RNaseP RNA (Kazantsev *et al.*, 2011 ▶). The SAXS experiments from SIBYLS are high quality and provide powerful experimental observations that constrain hypotheses and validate computational models of the solution state.

A significant advance made at SIBYLS was the determination that biomolecular SAXS experiments can distinguish between flexibility resulting from discreet conformational switching and domain delocalization (Rambo & Tainer, 2011 ▶). SAXS can help answer key biological questions about how flexible and unstructured regions avoid toxic mis-assemblies, and may explain how mutations that disrupt the stability of enzymes, such as seen for superoxide dismutase and XPD (Perry *et al.*, 2010 ▶; Didonato *et al.*, 2003 ▶; Fan *et al.*, 2008 ▶; Shin *et al.*, 2009 ▶), are tolerated in the cells.

From a functional point of view, flexibility can endow a protein with thermodynamic properties that are beneficial for achieving specificity. For example, the interaction sites of RPA for ssDNA at the flap junction exploit the enhanced flexibility of ssDNA to differentiate between dsDNA (Pretto *et al.*, 2010 ▶). In combination with computational modeling, SAXS has been used to establish multiple conformations for the post-translation modification of ubiquitin on PCNA, providing an understanding of its biological effects (Tsutakawa, Van Wynsberghe *et al.*, 2011 ▶). The combination of SAXS with biochemistry also revealed the solution structure for a human mismatch repair complex on DNA loops associated with human degenerative disease (Lang *et al.*, 2011 ▶). For ATPases, SAXS results not only established assembly state conformations, but also established how ligands can regulate and increase activity, as seen for the stimulation of the flagella ATPase motor by specific lipids (Ghosh *et al.*, 2011 ▶). These and other results demonstrate that SAXS data can provide insight and address unanswered biological questions.

### 8.2. MX results

Crystal structures determined at the SIBYLS beamline have defined details of active site water molecules in the DNA repair nuclease EndoIV (Garcin *et al.*, 2008 ▶; Ivanov *et al.*, 2007 ▶) and in the replication and repair flap endonuclease FEN1 (Tsutakawa, Classen *et al.*, 2011 ▶), providing insights into unified enzyme mechanisms. As metalloproteins are largely uncharacterized even in microbial metalloproteomes (Cvetkovic *et al.*, 2010 ▶), the structural characterization of metal ion binding sites has been an important focus area for users of the SIBYLS beamline, including the investigation of toxic metal ions, such as cadmium, and their role in mismatch repair inhibition (McMurray & Tainer, 2003 ▶). SIBYLS MX analyses of metalloprotein binding sites have also revealed novel domains and activities, as seen for the iron–sulfur cluster domain in XPD helicase, which acts in DNA repair and transcription (Fan *et al.*, 2008 ▶), and the non-heme hexameric Ni superoxide dismutase (NiSOD) complex (Barondeau *et al.*, 2004 ▶).

Another area with exemplifying biological discoveries has concerned complexes and post-translation modifications that are underrepresented in the Protein Data Bank (PDB; Berman *et al.*, 2000 ▶), such as the BRC1 protein complex with phosphorylated histone H2A at 1.45 Å resolution (Williams, Williams *et al.*, 2010 ▶). Whole classes of post-translation modifications are controlled by similar distinct non-covalent complexes as shown for SUMO and ubiquitin modifications (Prudden *et al.*, 2011 ▶; Heideker *et al.*, 2011 ▶).

The SIBYLS beamline has provided several biologically informative DNA and RNA structures and complexes that have helped flesh out another underrepresented area in the PDB. For example, a single unrepaired alkylguanine in DNA can cause apoptosis. Structures of alkyltransferase-like (ATL) proteins with multiple alkyl–DNA complexes, determined at the SIBYLS beamline, revealed a new non-enzymatic base flipping mechanism for directing DNA repair pathway selection (Tubbs *et al.*, 2009 ▶). Lesion binding by ATL directs the repair response from base repair by a single protein to nucleotide excision repair that removes a patch of ∼30 bases and requires the coordination of helicases and nucleases (Fuss & Tainer, 2011 ▶).

Substrate, product and mutant structures of human FEN1–DNA complexes from the SIBYLS beamline helped solve a decades old puzzle of how FEN1 specifically incises ∼50 million Okazaki fragments during human DNA replication while enhancing the hydrolysis rate of phosphodiester bonds by ∼10^17^. Collectively, the multiple FEN1 structures provided a unified model involving ‘double-strand DNA binding, single-strand DNA incision’, that is likely to be a prototype for the entire 5′ nuclease superfamily (Tsutakawa, Classen *et al.*, 2011 ▶).

In general, the beamline is directed toward connecting structures to biological outcomes. Comparison of multiple structures has revealed flexibility and conformations relevant to specificity and activity (Kim *et al.*, 2012 ▶; Tsutakawa, Classen *et al.*, 2011 ▶). Indeed, MX experiments at SIBYLS have aided the design of improved photosensors that decrease the flexibility of the bound chromophore environment (Christie, Hitomi *et al.*, 2012 ▶) and revealed conformations for receptor binding and signaling (Christie, Arvai *et al.*, 2012 ▶; Nishimura *et al.*, 2009 ▶). High-resolution structures of assemblies have been critical to elucidate biological functions and guide chemical experiments on GFP, RFP and the iLov fluorescence biosensor (Christie, Hitomi *et al.*, 2012 ▶; Barondeau, Kassmann *et al.*, 2006 ▶; Barondeau, Tainer & Getzoff, 2006 ▶; Tubbs *et al.*, 2005 ▶). Additional high-resolution studies enabled by the SIBYLS beamline include details of SOD oxidized and reduced states forming a basis for reactive oxygen controls (Shin *et al.*, 2009 ▶; Barondeau *et al.*, 2004 ▶; Hearn *et al.*, 2004 ▶), molecular mimicry of SUMO (Prudden *et al.*, 2009 ▶), and damaged DNA binding and incision by endonuclease IV (Garcin *et al.*, 2008 ▶).

### 8.3. Combined SAXS and MX results

Conformational changes in macromolecules include disorder to order transitions, plastic deformations and domain rotations. Several publications from SIBYLS users show that SAXS coupled to MX is a particularly powerful means to experimentally characterize these conformational states. For example, in the XLF–XRCC4 complexes acting in the repair of DNA breaks, SAXS revealed filaments that promote ligation of blunt-ended DNA (Hammel, Yu, Fang *et al.*, 2010 ▶). Underscoring a synergy of SAXS and MX, these SAXS results characterized the assembly but also suggested constructs for crystallization that ultimately led to the crystal structure, which furthermore validated the prior SAXS assembly model (Hammel *et al.*, 2011 ▶). The combined results provided a unified model for DNA end joining *via* a dynamic scaffolding assembly.

Combined SAXS and MX results also revealed that DNA ligase binds to the replication processivity factor PCNA in an extended conformation that then wraps around DNA like a molecular watch band, providing insights on efficient localization and DNA loading (Pascal *et al.*, 2006 ▶). Furthermore, SAXS and MX results from human ligase III revealed dynamic switching between DNA bound states that accounts for its ability to ligate blunt DNA ends (Cotner-Gohara *et al.*, 2010 ▶). Differential conformational stability of protein–DNA complexes provides a basis for coordinating replication *versus* repair pathways as seen for FEN1 (Querol-Audí *et al.*, 2012 ▶). Hybrid MX–SAXS results were critical for elucidating how interactions that favor a given partner conformation can control biological outcomes: as observed for the clamp protein PCNA interactions where the function of the clamp loader (replication factor C) is dependent on the selective stabilization of the open conformation of the clamp (Tainer *et al.*, 2010 ▶).

Hybrid MX–SAXS results show how conformations and assemblies provide regulation for kinases (Rosenberg *et al.*, 2005 ▶; Min *et al.*, 2009 ▶). For ATPases, combined results changed our understanding of the superfamily ATPase, which acts in bacterial secretion and filament assembly for motility (Yamagata & Tainer, 2007 ▶). SAXS and MX results also suggest opening for substrate binding and closing for catalysis as a general theme for many systems, including proteases such as the M16 family of zinc peptidases, where clam-shell closure is required for proteolytic activity (Aleshin *et al.*, 2009 ▶).

A powerful application of SAXS is to validate the solution assembly state (Chayen *et al.*, 2003 ▶), as was done for the relatively small but functionally important interface in the nuclease that acts in DNA strand break repair (Williams *et al.*, 2008 ▶). It is often quite useful to show experimentally that the MX structure is identical to the SAXS solution structure, as seen, for example, for Bcr1 bound to phosphorylated histone (Williams, Williams *et al.*, 2010 ▶). For the UV photosensor Uvr8, hybrid MX–SAXS results revealed the correct solution assembly and revealed how UV photoabsorption by tryptophan promotes dimer dissociation and signaling (Christie, Arvai *et al.*, 2012 ▶). For the absisic acid drought receptor, SAXS plus MX identified the correct solution state dimer and the basis for receptor function (Nishimura *et al.*, 2009 ▶).

During DNA repair, intermediates are protected by tight product binding and by direct handoff from one repair step to the next (Hitomi *et al.*, 2007 ▶). For such DNA repair proteins several combined MX–SAXS results have demonstrated how flexible extensions recruit partner proteins to the site of DNA damage while a core DNA damage recognition domain remains tightly bound at the damaged site (Williams *et al.*, 2008 ▶, 2009 ▶; Pretto *et al.*, 2010 ▶; Cotner-Gohara *et al.*, 2010 ▶; Hammel, Yu, Mahaney *et al.*, 2010 ▶).

For the Mre11 nuclease, Rad50 ATPase and Nbs1 binding protein (MRN complex) with roles in replication fork processing and double-strand break repair, MX plus SAXS results show that various MRN conformations control sensing, signaling and effector responses (Williams *et al.*, 2009 ▶; Williams *et al.*, 2011 ▶). The combinations of multiple complexes and conformational states allow the MRN complex to integrate information on the cellular state and affect optimal outcomes appropriate to cell and DNA status (Williams, Lees-Miller & Tainer, 2010 ▶).

## 9. Conclusions

Our knowledge of the nature of macromolecular function has been substantially advanced by major progress in two distinct areas, both supported efficiently by the SIBYLS beamline: (1) the ability to obtain high-resolution detail from MX coupled to (2) experimental data on flexibility. Although objective quantitative experimental measures of flexibility and disorder in solution are limited, SAXS provides a good assessment of macromolecular flexibility, shape and assembly. Combined MX and SAXS provide superb opportunities to examine the interface of biological networks such as replication and repair (Grasby *et al.*, 2012 ▶) and replication and transcription (Fuss & Tainer, 2011 ▶).

To summarize, we have presented the major technical features and capabilities of the SAXS and MX endstations of the SIBYLS beamline at the Advanced Light Source. With the advent of X-ray free electron lasers such as SLAC’s Linac Coherent Light Source (Emma *et al.*, 2010 ▶) and Lawrence Berkeley National Laboratory’s Next Generation Light Source a premium will be placed on available endstations. The SIBYLS beamline with its rapidly and easily reconfigurable endstations may serve as an example of how such valuable resources can be shared between different experimental techniques. Multi-purpose beamlines and endstations are often assumed to require compromises that reduce the functionality of the individual purposes. Our experience suggests that with thoughtful design the opposite can also be true. The dual endstation SIBYLS beamline promotes innovations in technology and software for MX and SAXS, and offers opportunities for staff to do better science and provide more effective user support. The future of structural biology lies not in the determination of the structures of isolated individual proteins or other macromolecules, but in the understanding of the dynamic, flexible and interwoven nature of the interactions between macromolecules. By combining two powerful techniques at one beamline our vision has been to constantly promote the use of hybrid methods to probe the difficult questions of biology.

## Supplementary Material

Supplementary material file. DOI: 10.1107/S0021889812048698/he5572sup1.txt
Oxygen-free verification: experimental details

## Figures and Tables

**Table:** 

Beamline name	SIBYLS
X-ray source	5 T superbend
Source size (r.m.s., H × V, µm)	230 × 30
Source divergence (r.m.s., H × V, mrad)	1.5 × 0.5
Mirrors	M1 Rh/Pt coated INVAR
	M2 Rh/Pt coated Si toroid
Monochromator	Double-crystal Si(111) or Mo/B_4_C multilayer
Energy resolution: Si(111) (*E*/Δ*E*)	7000
Energy resolution: Mo/B_4_C multilayer (*E*/Δ*E*)	110
Demagnification ratio	2:1
Wavelength range (Å)	0.73–2.5

**Table:** MX endstation.

Beam size (collimated) (µm)	Variable from 20–120
Beam size (uncollimated) (µm)	165 × 130
Typical exposure time (s)	0.5–5.0
Flux (collimated, 100 µm, 500 mA, photons per second)	2 × 10^11^
Sample automation	DOMO (SSRL-Style SAM Automounter)
Goniometry	Fox air bearing with Huber XYZ sample stage
X-ray detector type	Fiber-optic coupled CCD
X-ray detector model	ADSC Quantum 315r
2θ capabilities (°)	−5 to +45

**Table:** SAXS endstation.

X-ray detector type	Fiber-optic coupled CCD
X-ray detector model	mar165 (now Rayonix)
Sample format	∼15 µl of solution in 96-well plates
Sample environment (K)	269–353
Sample automation	Hamilton liquid-handling robot (288-sample capacity)
Beam size (at sample) (mm)	5.0 × 0.5
Flux (uncollimated, 500 mA, photons per second)	2 × 10^13^

## Footnotes

1 Supplementary information for this paper is available from the IUCr electronic archives (Reference: HE5572). Services for accessing this material are described at the back of the journal.
